# A calreticulin-linked HPV-16 E7 minigene DNA vaccine elicits strong E7-specific CD8+ T-cell immunity and durable antitumor effects in a preclinical model

**DOI:** 10.1038/s41598-026-58415-z

**Published:** 2026-07-03

**Authors:** Yu-Cheng Chang, Yichu Xu, Ya-Chea Tsai, Ching-Wen Yu, Chih-Long Chang, Chuan-Hsiang Huang, T.-C. Wu, Chien-Fu Hung

**Affiliations:** 1 Department of Pathology, Johns Hopkins University School of Medicine, 1550 Orleans Street, CRB II 307, Baltimore, MD 21287 USA; 2 Department of Oncology, Johns Hopkins University School of Medicine, 1550 Orleans Street, CRB II 307, Baltimore, MD 21287 USA; 3 Department of Cell Biology, Johns Hopkins University, Baltimore, MD 21287 USA; 4 Department of Obstetrics and Gynecology, Johns Hopkins University School of Medicine, Baltimore, MD USA; 5 Molecular Microbiology and Immunology, Johns Hopkins University School of Medicine, Baltimore, MD USA; 6 Department of Medicine, MacKay Medical University, New Taipei City, Taiwan; 7 Department of Obstetrics and Gynecology, MacKay Memorial Hospital, Taipei, 104217 Taiwan

**Keywords:** DNA vaccine, Calreticulin, MHC trafficking domain, Ubiquitin, Cancer, Immunotherapy, Cancer, Immunology, Oncology

## Abstract

Calreticulin (CRT) is an endoplasmic reticulum chaperone that facilitates antigen processing and presentation, making it an attractive fusion partner for enhancing tumor-specific T-cell responses in DNA vaccine development. However, the relative efficacy of CRT compared with other antigen-presentation–enhancing strategies has not been fully evaluated. We constructed a DNA vaccine encoding the immunodominant human papillomavirus (HPV)-16 E7 minigene epitope (aa 49–57) fused to the C-terminus of CRT (pcDNA3-CRT-E7(49–57), hereafter CRT-E7(49–57)). This vaccine was compared with DNA vaccines encoding CRT linked to full-length E7 (CRT-E7(1–98)) and other antigen-presentation–enhancing constructs, including ubiquitin linked to E7 minigene (Ub-E7(49–57)) and MHC class I trafficking domain linked to E7 minigene (MITD-E7 (49–57)). We evaluated E7-specific cytotoxic T-cell responses, tumor volume, and survival in both prophylactic and therapeutic TC-1 tumor models. Intramuscular electroporation of C57BL/6 mice with CRT-E7(49–57) elicited robust E7-specific CD8⁺ T-cell responses comparable to those induced by CRT-E7(1–98) and MITD-E7(49–57), but higher than those elicited by Ub-E7 (49–57). CRT-E7(49–57) vaccination provided complete protection against TC-1 tumor challenge, with mice remaining tumor-free and surviving beyond 60 days. Vaccinated mice also exhibited strong immune memory, as tumor rechallenge triggered potent E7-specific CD8⁺ T-cell responses and restricted tumor growth. In therapeutic settings, two doses of CRT-E7(49–57) completely suppressed tumor progression, outperforming Ub-E7(49–57) and conferring superior survival compared with MITD-E7(49–57). The CRT-E7(49–57) DNA vaccine induces durable, high-magnitude E7-specific CD8⁺ T-cell responses and confers effective prophylactic and therapeutic antitumor immunity, underscoring its promise as a versatile platform for cancer immunotherapy.

**Supplementary Information:**

The online version contains supplementary material available at https://doi.org/10.1038/s41598-026-58415-z.

## Introduction

Calreticulin (CRT) is a 46 kDa, calcium-binding chaperone protein primarily found in the endoplasmic reticulum (ER) owing to the ER-retention signal KDEL located at its C-terminus^1–3^. CRT is a key component of the peptide loading complex (PLC) and plays a crucial role in antigen processing. It assists in stabilizing PLC, folding and stabilization of tapasin, retrieving sub-optimally folded major histocompatibility complex (MHC) class I (MHC I) from post-ER compartments, and ultimately leads to efficient presentation of peptide-MHC complexes^4–7^. Through its role in the quality control of MHC assembly, CRT allows robust adaptive immune responses against infections and cancer.

A significant effort has been devoted to targeting tumor-associated antigens (TAAs), which are present at low levels in normal cells but overexpressed in cancers. Recently, there has been growing interest in targeting tumor-specific antigens (TSAs), which are only expressed in cancer cells. Neoantigens, resulting from somatic gene mutations, are the most commonly targeted among TSAs^8^. Because neoantigens are completely absent in normal cells, they can circumvent thymus-negative selection, a process that eliminates T cells with strong affinity for self-antigens^9,10^. TAAs, due to their low-level expression in normal tissues as self-antigens, are subject to this tolerance mechanism. As a result, neoantigens tend to be more immunogenic than TAAs and represent a particularly promising target for personalized cancer immunotherapy. Advances in computational algorithms, such as next-generation sequencing for predicting MHC-binding epitopes, have greatly facilitated the identification of potentially immunogenic neoantigens^11,12^. In this context, fusing CRT with neoantigens offers a way for inducing robust T cell responses through the DNA vaccine platform, as CRT can deliver neoantigens directly to the ER for processing. One of the key advantages of DNA vaccines is their ability to co-encode multiple antigens along with molecules that can enhance the immunogenicity, such as CRT, within a single plasmid. In addition to their co-encoding capability, DNA vaccines are easy to produce, effective and cost-effective, stable during storage, and adaptable for personalization, making them an attractive formulation for cancer immunotherapy^13–15^. Several DNA vaccines have entered clinical trials^16,17^. For example, GX-188E, a DNA cancer vaccine targeting the E6 and E7 oncoproteins of human papillomavirus (HPV)-16 and HPV-18, was administered to patients with HPV-positive advanced cervical cancer and demonstrated a favorable safety profile^18^. Objective remission was achieved in 19 out of 60 patients, resulting in an overall response rate of 31.7%, suggesting favorable antitumor activity^18^.

We previously demonstrated that C57BL/6 mice vaccinated intradermally with pcDNA3-CRT-E7(aa1-98) exhibited a significant increase in E7-specific CD8⁺ T cell precursors and a potent antitumor effect against E7-expressing tumors compared to pcDNA3-E7(aa1-98) and pcDNA3-CRT groups^19^. The results highlight the effectiveness of CRT as a fusion partner, providing an excellent platform for antigen presentation. However, delivering full-length antigen relies on intracellular antigen processing like proteasome degradation, which may not consistently generate the desired epitope for presentation, and thus compromise the immune responses^20,21^. Processing of the original antigen may lead to immunodominant epitopes with high MHC affinity, some of which may fail to elicit effective responses if tolerance mechanisms or thymic deletion have eliminated the corresponding T-cell clones^22^. In contrast, epitope-specific vaccines may enhance MHC presentation efficiency by reducing the dependence on variable intracellular processing, making it a better option than the entire antigen^23^. Furthermore, replacing full-length E7 with epitope-specific E7 allows for the design of polyepitope vaccines, in which multiple distinct epitopes can be incorporated to elicit diverse T cell-mediated responses targeting multiple tumor-specific mutations^24,25^. This approach is limited when using full-length antigens largely due to size constraints. Therefore, we hypothesized that when combined with a novel CRT-based tumor treatment strategy, using epitope-specific pcDNA3-CRT-E7(aa49-57) instead of pcDNA3-CRT-E7(aa1-98) could enhance immunological responses.

Several other strategies have been employed to strengthen immune responses^26^, including synthetic long peptides^27–29^, exosome delivery^30–33^, ER chaperone fusion^34^, ubiquitination^35,36^, and MHC class I trafficking domain (MITD) fusion^37–39^. Ubiquitination is a reversible covalent post-translational modification that can regulate diverse cellular processes and is best known for targeting proteins for proteasomal degradation^40^. This degradation is a critical step in generating neoantigen peptides before their presentation on MHC molecules^41^. Fusing ubiquitin (Ub) sequences to antigens in DNA vaccines accelerates their degradation, thus enhancing antigen processing and presentation through the MHC I pathway^42^. Interestingly, this strategy has not been consistently effective in provoking antigen specific CD8^+^ T cell responses^40,43,44^. Nevertheless, the ubiquitination technique has been applied to the development of various polyepitope therapeutic vaccines^24^. Another approach often utilized in nucleic acid vaccine generation is fusing antigens to MITD, a sequence derived from the cytosolic domains of MHC I molecules. Fusing MITD to either minigene or full-length antigens promotes efficient peptide loading onto both MHC I and MHC class II molecules in murine and human dendritic cells, resulting in robust antitumor responses^37,38^. The strategy has shown encouraging results in generating polyepitope vaccines in humans^39,45^. To better evaluate the efficacy of our approach, we compared the ubiquitination and MITD strategies with our CRT-fusion construct in terms of their anticancer activity.

## Methods

### Plasmid DNA constructs and preparation

The generation of pcDNA3-CRT^19^, pcDNA3-CRT-E7(1–98)^19^, and pcDNA3-E7(49–57)^46^ has been described previously. To generate pcDNA3-CRT-E7(49–57), which encodes the HPV16 E7 amino acids (aa) 49–57 epitope, synthetic oligonucleotides (AATTCATGAGAGCCCATTACAATATTGTAACCTTTTAAA and AGCTTTTAAAAGGTTACAATATTGTAATGGGCTCTCATG) were inserted into the EcoRI and HindIII restriction sites of pcDNA3-CRT. pcDNA3-1Ub-E7(49–57), encoding a fusion protein consisting of one copy of mutated ubiquitin (G76V)^47^ linked to the HPV16 E7 aa 49–57 epitope, was generated using a synthesized sequence (GenScript Corporation, Piscataway, NJ) cloned into the pcDNA3.1 vector. pcDNA3-4Ub-E7(49–57), encoding four tandem repeats of mutated ubiquitin (G76V) fused to the HPV16 E7 aa 49–57 epitope, was also synthesized by GenScript Corporation and cloned into pcDNA3.1. For pcDNA3-MITD-E7(49–57), the sequence encoding MITD-E7(49–57) was synthesized by GenScript Corporation and cloned into pcDNA3.1. This construct includes an MHC class I signal peptide (SP), the HPV16 E7 aa 49–57 epitope, and the transmembrane and cytosolic domains of MITD^38^. Plasmid DNA was prepared using the HiSpeed Plasmid Maxi Kit (Qiagen), with purity assessed by a Nanodrop instrument with an A260/A280 ratio of approximately 1.8. The preparation contained > 90% supercoiled DNA with endotoxin levels of approximately 10 EU/µg.

### Cell culture

HPV16 E6- and E7-expressing TC-1 cells were generated as previously described^48^. DC2.4 dendritic cells^49,50^ were described previously. Both cell types were maintained in RPMI medium supplemented with 2 mM glutamine, 1 mM sodium pyruvate, 100 IU/mL penicillin, 100 µg/mL streptomycin, and 10% fetal bovine serum (FBS). The generation of 293 cells expressing the murine MHC class I molecule Db (293-Db cells) was described previously^51^. The establishment of the murine HPV16 E7 peptide (aa 49 to 57)-specific CD8 + T cell line was described previously^50^. Briefly, E7-specific CD8⁺ splenocytes were isolated from mice vaccinated with E7 DNA and subsequently co-cultured with E7-expressing target cells. These cells were maintained in culture with repeated antigen stimulation under conditions that support antigen-specific expansion, resulting in the enrichment of E7-specific CD8⁺ T cells.

### In vitro HPV16 E7 antigen presentation assay

To assess the ability of transfected cells to present HPV16 E7, DC2.4 cells were transfected with 1 µg of one of the following constructs: (1) empty pcDNA3 vector, (2) pcDNA3-1Ub-E7(49–57), (3) pcDNA3-4Ub-E7(49–57), (4) pcDNA3-E7(49–57), or (5) pcDNA3-CRT-E7(49–57). After 24 h when transfection efficiency reached approximately 60%, 2.5 × 10^5^ transfected cells were collected and co-cultured with an equal number of HPV16 E7 (amino acids 49–57)-specific CD8 + T cells in 96-well round-bottom plates. GolgiPlug (BD Pharmingen, San Diego, CA) was added to the culture for a period of 20–24 h. Following incubation, cells were stained for intracellular IFN-γ and analyzed by flow cytometry (Beckman Coulter CytoFLEX S) to evaluate HPV16 E7-specific CD8 + T cell activation. Details of the flow cytometry gating strategy are provided in Supplementary Fig. 1.

### Mice

Female C57BL/6 mice aged 6–8 weeks were purchased from Charles Rivers Laboratories (Frederick, Maryland, USA) and housed under specific pathogen-free conditions at the Johns Hopkins University School of Medicine Animal Facility (Baltimore, Maryland, USA). All animal procedures were performed according to protocols approved by the Johns Hopkins Institutional Animal Care and Use Committee. Recommendations for the proper use and care of laboratory animals were closely followed, and all relevant guidelines and regulations were met as experiments were performed. Euthanasia was performed by CO_2_ asphyxiation as per Oncology Center routine in accordance with the Panel on Euthanasia of the American Veterinary Medical Association. Death is verified by verification that heart has stopped beating and by observation that the animal fails to recover within 10 min after CO_2_ exposure ends.

### Electroporation-mediated DNA vaccination

For intramuscular vaccine injection with electroporation, plasmids were prepared and injected using a 1 mL syringe equipped with a 31-gauge needle into the tibialis muscle of shaved hind legs of mice followed by electroporation with an ECM830 Square Wave Electroporation System (BTX Harvard Apparatus company, Holliston, MA, USA). Each leg received 5 µg of the vaccine in 30 µL of PBS. The electroporator was set to deliver 8 pulses at 106 V with a 20 ms pulse duration and 200 ms interval. Booster vaccination(s) were administered using the same dose and regimen as the priming vaccination.

### Tetramer staining to detect presence of E7-specific CD8 + T cells

Blood samples were obtained from mice through facial vein lancet puncture and collected into tubes containing 100 µL of EDTA (0.5 M, pH 8). PBMCs were prepared by lysing red blood cells with RBC lysis buffer (eBioscience) followed by filtration through a cell strainer plate. Prior to antibody labeling, cells were incubated with Zombie Aqua viability dye (Fixable Viability Kit) to discriminate between live and dead cells, and Fc Block was applied to reduce nonspecific antibody interactions. E7-specific CD8⁺ T cells within the PBMCs were detected by staining with PE-labeled E7 tetramer (1:200) and APC-A750-conjugated anti-mouse CD8α antibody (1:1000) for 30 min at 4 °C. After wash, samples were run on a Beckman Coulter CytoFLEX S flow cytometer, and data were processed using FlowJo version 10.4 (FlowJo LLC). Details of the flow cytometry gating strategy are provided in Supplementary Fig. 1.

### In vivo tumor protection and treatment experiments

For prophylactic models, to compare the protective efficacy of CRT-E7(49–57) and CRT-E7(1–98), (on day 1) mice were immunized with 10 µg of designated vaccine via intramuscular injection followed by electroporation. Two booster immunizations were administered on days 8 and 28. Four weeks after the final vaccination, mice were subcutaneously challenged with 1 × 10⁵ TC-1 cells in the left flank. To assess the long-term protective efficacy of the vaccine, mice were subcutaneously rechallenged with same number of TC-1 cells 60 days after the initial tumor challenge using the same regimen. To compare the protective efficacy of CRT-E7(49–57) with other established strategies, same vaccination schedule was used except only one booster was administered. 1 × 10⁵ TC-1 cells were implanted three weeks after the final vaccination in the left flank.

For therapeutic models, mice were subcutaneously challenged with 1 × 10⁵ TC-1 cells per mouse. Four days after tumor challenge, the mice received a 10-µg intramuscular prime immunization with designated vaccines, followed by electroporation. Booster immunizations using the same regimen were administered at one and two weeks after the initial immunization.

For all models, survival was monitored for 60 days following the tumor challenge. Tumor development was assessed weekly by palpation, and tumor size was measured with a digital caliper. Tumor volume (mm^3^) was calculated using the formula: length × length × width × 0.5. General anesthesia was induced via intraperitoneal injection of a cocktail containing Ketamine (80 mg/kg) and Xylazine (10 mg/kg). The solution was administered using a 1 mL syringe equipped with a 25-gauge needle. Mice were euthanized once tumor diameter exceeded 1.7 cm when other humane endpoints defined by the Johns Hopkins Animal Care and Use Committee were reached, at which point they were considered to have succumbed to tumor burden. Euthanasia was performed by CO_2_ asphyxiation as per Oncology Center routine in accordance with the Panel on Euthanasia of the American Veterinary Medical Association. Death was confirmed by cessation of cardiac activity and absence of recovery within 10 min following CO_2_ exposure.

### Statistical analysis

All data are expressed as mean ± standard deviation (S.D). Kaplan-Meier survival curves were generated to evaluate survival rates and log-rank tests were performed to compare survival time between treatment groups. The statistical significance is determined by one-way ANOVA with Tukey-Kramer multiple comparison, two-way ANOVA with the Geisser-Greenhouse correction or Student’s t test using the GraphPad Prism 10 software (GraphPad, CA, USA). All *p* ≤ .05 were considered significant (*p < .05; **p < .01; ***p < .001; ****p < .0001).

## Results

### DNA vaccine encoding CRT and E7-specific immunodominant CTL epitope CRT-E7(49–57) generates potent immune response and protective effect against TC-1 tumors

**Fig. 1:**
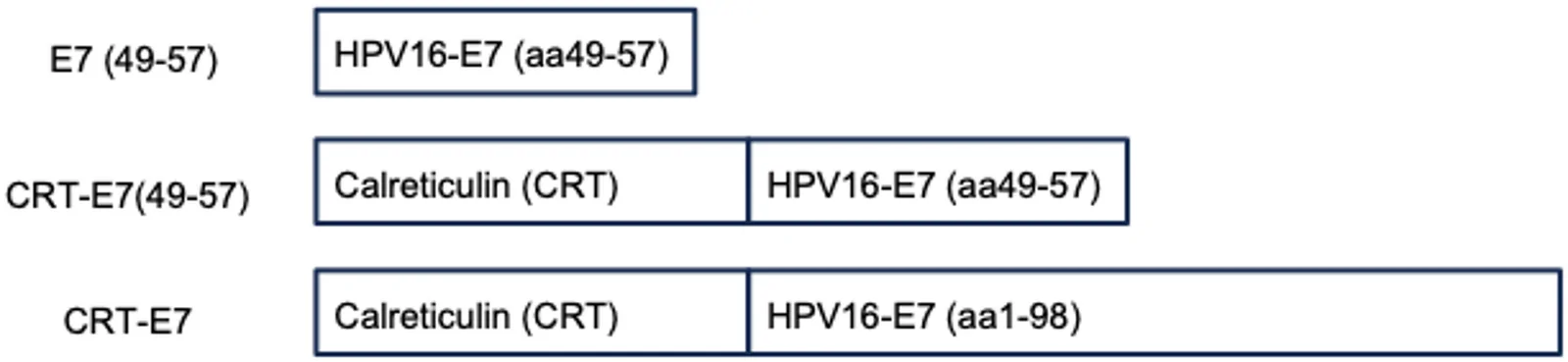
DNA construct of pcDNA3-E7(49–57), pcDNA3-CRT-E7(1–98), and pcDNA3-CRT-E7(49–57). The HPV16 E7 CTL epitope (aa49-57) was cloned into the pcDNA3 vector to produce the pcDNA3-E7(49–57). The HPV16 E7 epitope (aa49-57) and full sequence (aa1-98) were each individually fused to the C-terminus of the full-length CRT and then cloned into the pcDNA3 vector to generate the fusion constructs pcDNA3-CRT-E7(49–57) and pcDNA3-CRT-E7(1–98), respectively.

**Fig. 2:**
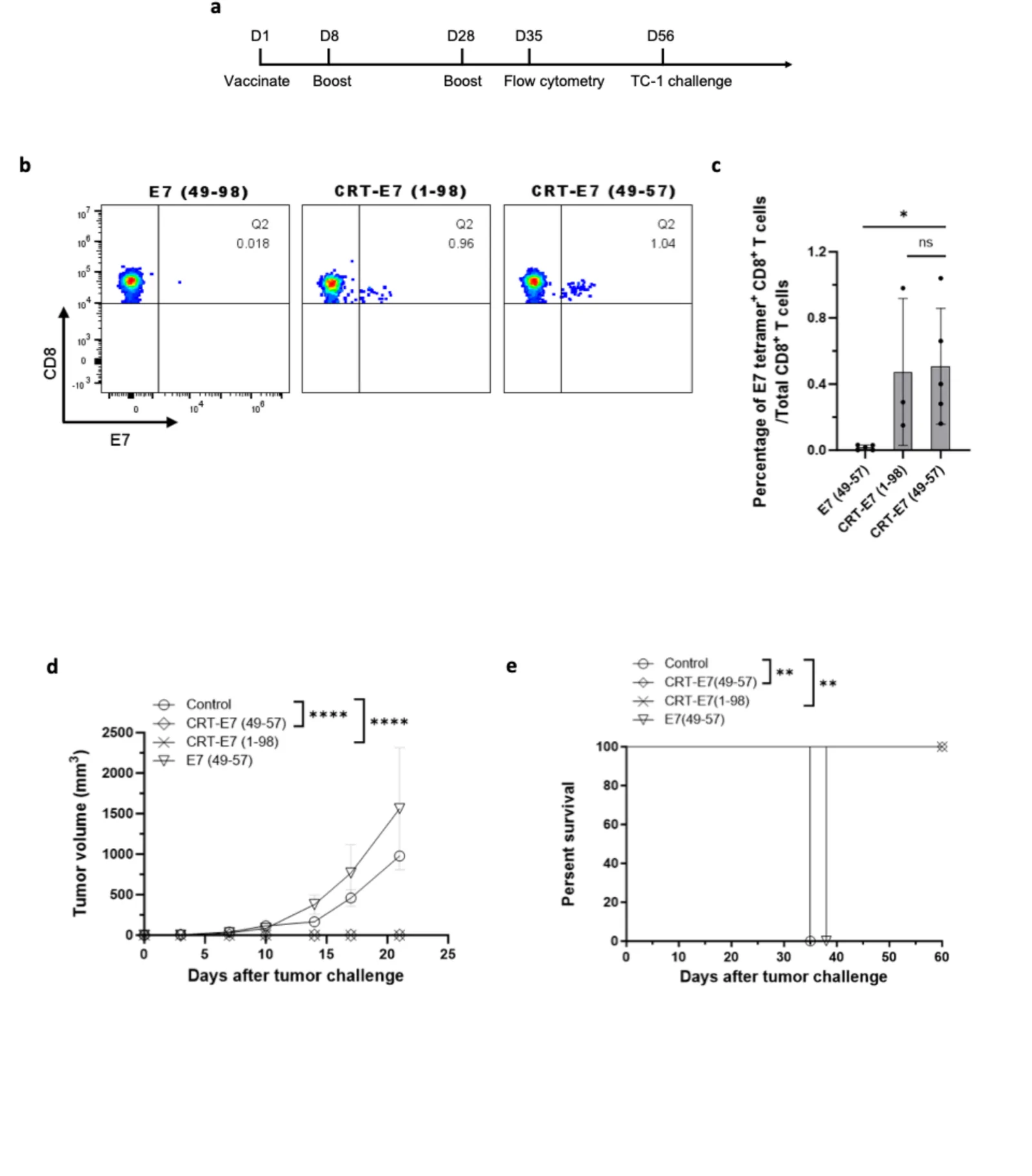
In vivo tumor protection experiments a Timeline of the experiment. Briefly, on day 1, mice were immunized intramuscularly with (1) 10 µg of pcDNA3-CRT-E7(49–57) (*n* = 5), (2) 10 µg of pcDNA3-CRT-E7(1–98) (*n* = 3), or (3) 10 µg of pcDNA-E7(49–57) (*n* = 5), followed by electroporation. On days 8 and 28, the mice were boosted using the same regimen. On day 35, PBMCs were collected and lymphocytes were analyzed via flow cytometry. On day 56, mice were subcutaneously challenged with 1 × 10^5^ TC-1 tumor cells. (**a**) Tumor size and survival were subsequently monitored. (**b**) Representative flow cytometry plot showing the frequency of E7-specific CD8⁺ T cells within the CD8⁺ T cell population. (**c**) Bar graph summarizing the quantitative results of the flow cytometry analysis. (**d**) Line graph illustrating tumor volume progression in TC-1 tumor-challenged mice. (**e**) Kaplan-Meier analysis was used to assess the 60-day survival of mice bearing TC-1 tumors (*n* = 5 for control). The sharp drop from 100% to 0% is likely because of the small sample size and the clustered timing of tumor progression. Data are presented as mean ± SD and analyzed by one-way ANOVA (**c**), repeated measures ANOVA (**d**), and Kaplan-Meier method with Log-rank (Mantel-Cox) test (**e**). *p < .05, **p < .01, ****p < .0001, ns: not significant.

The full-length and epitope-specific CRT-E7 sequences were generated by fusing either the complete HPV16 E7 sequence (aa1-98) or its epitope (aa49-57) to the C-terminus of CRT (Fig. 1). To focus the immune response on a dominant cytotoxic T-cell determinant and enhance antigen presentation, the epitope-specific construct was designed as a minigene encoding only the immunodominant E7 peptide (aa49-57). This design reduces irrelevant sequences from the full-length antigen, improving processing and MHC I presentation efficiency. Each construct was then individually cloned into pcDNA3 to produce pcDNA3-CRT-E7(1–98) (full-length CRT-E7) and pcDNA3-CRT-E7(49–57) (epitope-specific CRT-E7). Likewise, pcDNA3-E7(49–57) (epitope-specific E7) was also generated via direct cloning into pcDNA3 without CRT fusion (Fig. 1). To assess the immunogenicity of the CRT-E7(49–57) minigene vaccine in vivo, mice were vaccinated intramuscularly in the hind leg with either CRT-E7(49–57), CRT-E7(1–98), or E7(49–57), followed by electroporation. Two boost doses were given within 28 days using the same regimen (Fig. 2a). Seven days after the last vaccination, peripheral blood mononuclear cells (PBMCs) were collected and analyzed via flow cytometry. The mice that were vaccinated with E7(49–57) exhibited a weak E7-specific CD8^+^ T cell response, whereas those vaccinated with either CRT-E7(49–57) or CRT-E7(1–98) had comparable and robust E7-specific CD8^+^ T cell responses (Fig. 2b, c). These results show that the CRT-E7(49–57) minigene vaccine effectively induced potent antigen-specific immune responses in vivo. To test the protective antitumor effect of the CRT-E7(49–57) minigene vaccine in vivo, mice were subcutaneously challenged with TC-1 tumor cells three weeks after the flow analysis (Fig. 2a), with unvaccinated, tumor-challenged mice serving as controls. Tumor volume and survival were monitored. The mice that received vaccination with either CRT-E7(49–57) or CRT-E7(1–98) exhibited no detectable tumor growth compared to mice in E7(49–57) and control group (Fig. 2d). Moreover, both CRT-E7-vaccinated (full-length and epitope-specific) mice survived significantly longer than other groups (Fig. 2e). These results demonstrate that the CRT-E7(49–57) vaccine efficacy induces potent antigen-specific immune responses in vivo.

### CRT-E7(49–57) provides durable protection against TC-1 tumors

**Fig. 3:**
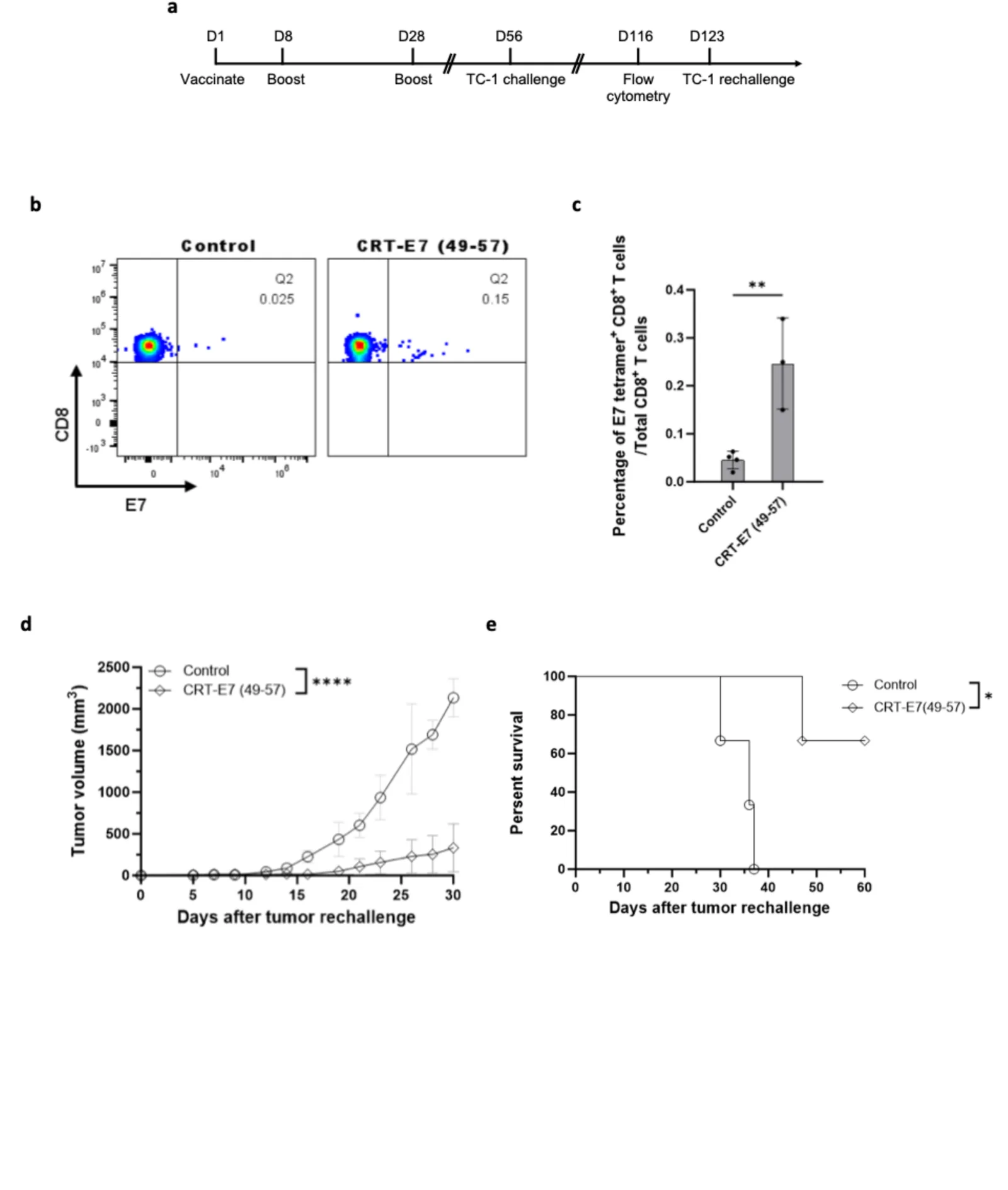
Evaluation of long-term immune memory response in mice treated with CRT-E7(49–57). (**a**) Timeline of the experiment. 60 days after the initial tumor challenge, lymphocytes from PBMCs were analyzed by flow cytometry (*n* = 4 for control; *n* = 3 for pcDNA3-CRT-E7(49–57)). Only 3 mice were included in the pcDNA3-CRT-E7(49–57) group because 2 mice developed tumors prior to analysis. Seven days after blood analysis, mice were rechallenged with 1 × 10^5^ TC-1 tumor cells. Tumor size and survival were monitored. (**b**) Representative flow cytometry plot illustrates the proportion of E7-specific CD8⁺ T cells among the CD8⁺ T cell population. (**c**) Bar graph presenting the results of the flow cytometry analysis. (**d**) Line graph showing tumor volume progression in TC-1 tumor-rechallenged mice. To ensure equal sample size between groups, three control mice were randomly selected for this and subsequent analysis. (**e**) Kaplan-Meier analysis was used to assess the 60-day survival of mice bearing rechallenged TC-1 tumors. Data are presented as mean ± SD and analyzed by one-way ANOVA (**c**), repeated measures ANOVA (**d**), and Kaplan-Meier method with Log-rank (Mantel-Cox) test (**e**). *p < .05, **p < .01, ****p < .0001.

We previously demonstrated that the CRT-E7(1–98) vaccine completely inhibited tumor growth by generating a strong E7-specific immune response in vivo^19^. In this study, the results showed that vaccination CRT-E7(49–57) achieved comparable survival outcomes. To assess the durability of the immune memory generated by this promising vaccine, PBMCs were collected from mice 60 days after tumor challenge and analyzed via flow cytometry. The vaccinated mice maintained a significant level of E7-specific CD8^+^ T cells, whereas the untreated control group exhibited drastically weaker responses (Fig. 3a, b). To evaluate whether the immune responses could confer sustained protection against TC-1 tumors, mice were rechallenged with TC-1 cells 67 days after the initial subcutaneous challenge using the same regimen. Mice receiving CRT-E7(49–57) had reduced tumor growth within 30 days and prolonged survival over the subsequent 60-day period (Fig. 3c, d). These results indicate that the CRT-E7(49–57) vaccine induces durable and robust E7-specific CD8^+^ T cell responses, offering long-lasting immune memory and effective tumor protection in vivo.

### CRT-E7(49–57) elicits potent therapeutic antitumor effects in mice with TC-1 tumors

**Fig. 4:**
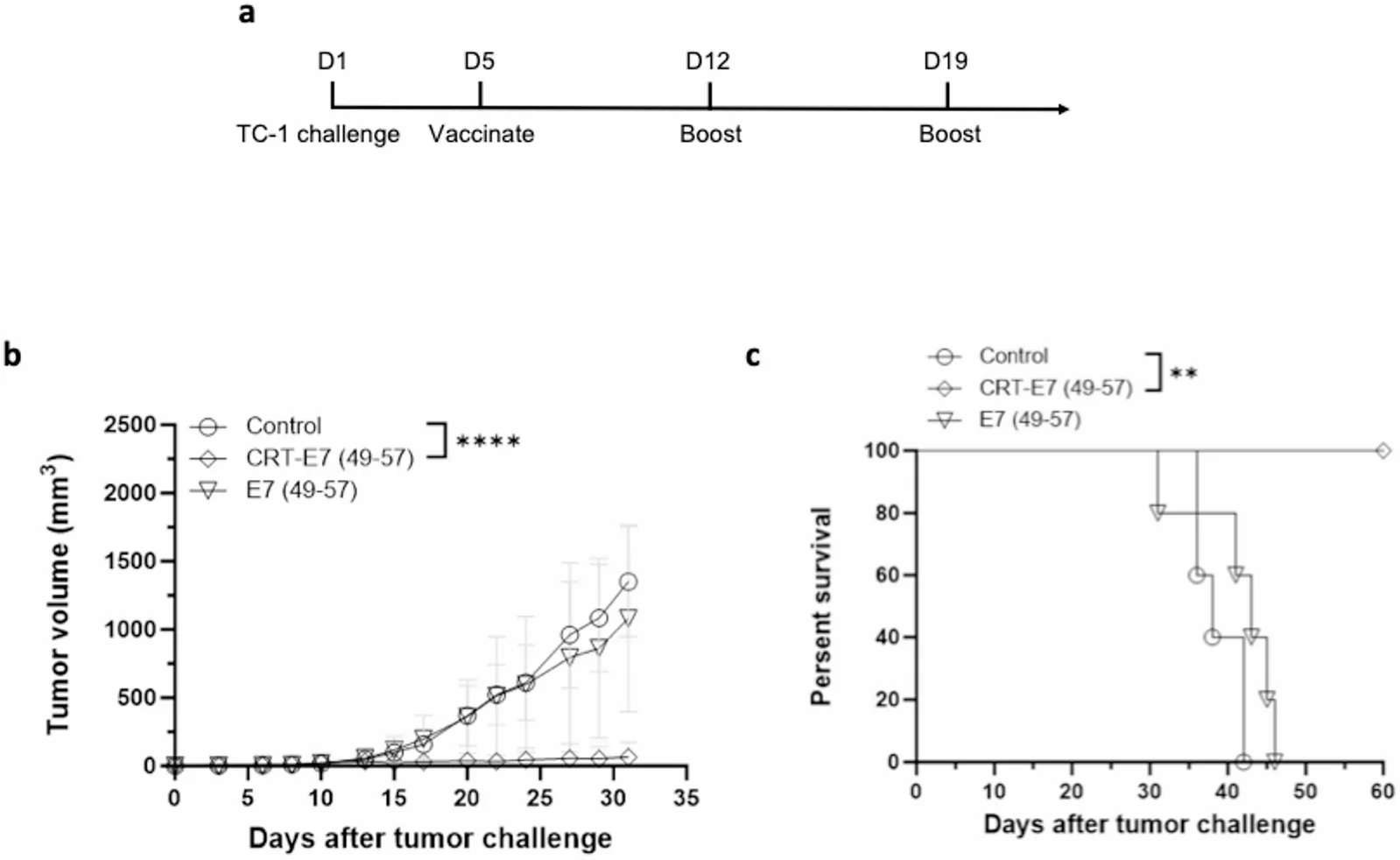
In vivo tumor treatment experiments using CRT-E7(49–57). (**a**) Timeline of experiment. Briefly, on day 1, Mice (*n* = 5 per group) were challenged subcutaneously with 1 × 10^5^ TC-1 tumor cells. On day 5, mice were immunized intramuscularly with (1) control, no treatment, (2) 10 µg of pcDNA3-CRT-E7(49–57), or (3) 10 µg of pcDNA3-E7 (49–57), followed by electroporation. On days 12 and 19, the mice received booster doses following the same regimen. Tumor size and survival were subsequently monitored. (**b**) Line graph illustrating tumor volume progression in TC-1 tumor-challenged mice. (**c**) Kaplan-Meier analysis was used to assess the 60-day survival of mice bearing TC-1 tumors. Data are presented as mean ± SD and analyzed by repeated measures ANOVA (**c**) and Kaplan-Meier method with Log-rank (Mantel-Cox) test (**c**). **p < .01, ****p < .0001.

Based on the findings that CRT-E7(49–57) could prophylactically protect mice against TC-1 tumor, we next investigated the therapeutic potential of CRT-E7(49–57) in mice bearing TC-1 tumors. Mice were challenged subcutaneously with TC-1 tumor cells on day 1, followed by leg intramuscular administration of CRT-E7(49–57) or E7(49–57) on day 5. Two booster doses were administered weekly after the initial vaccination using the same regimen (Fig. 4a). Mice treated with CRT-E7(49–57) developed significantly smaller tumors compared to both the untreated control group and the group receiving the E7(49–57) vaccine alone (Fig. 4b). Furthermore, all mice in the CRT-E7(49–57) group survived for 60 days, whereas those in the other groups died progressively, with none surviving past day 46 (Fig. 4c). We therefore believe that CRT-E7(49–57) vaccination could serve as an effective therapeutic strategy for controlling tumor progression and improving survival.

### CRT-E7(49–57) and MITD-E7(49–57) induce robust E7-specific immune responses and superior prophylactic efficacy compared to Ub-E7(49–57)

**Fig. 5:**
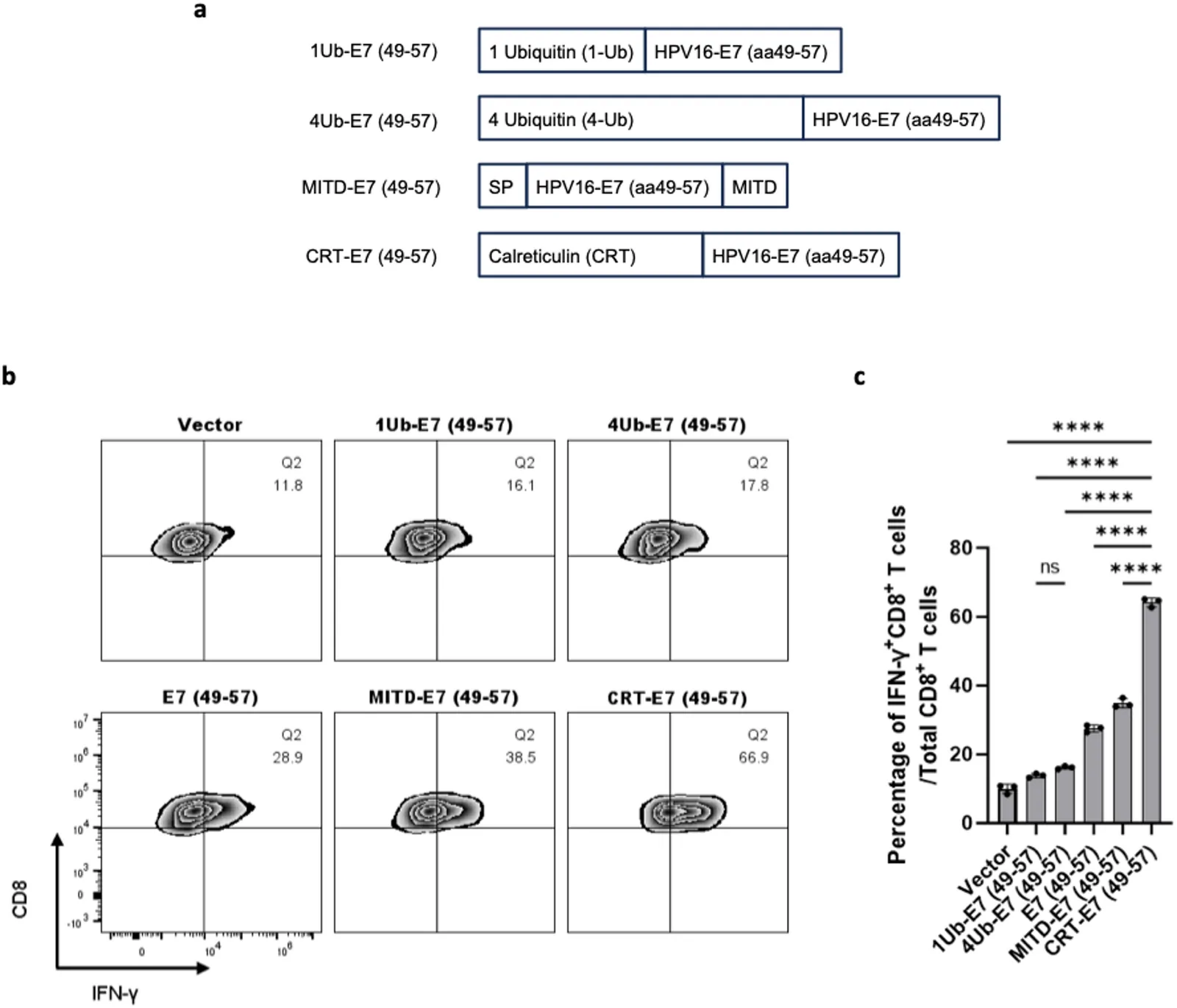
(**a**) DNA construct of pcDNA3-1Ub-E7(49–57), pcDNA3-4Ub-E7(49–57), pcDNA3-MITD-E7(49–57), and pcDNA3-CRT-E7(49–57). The HPV16 E7 epitope (aa49-57) was fused to the C-terminus of the full-length Ub (1Ub), four tandem-repeat of Ub (4Ub), or MITD (aa308-362, also includes an upstream signal peptide), and then each cloned into the pcDNA3 vector to generate the fusion constructs pcDNA3-1Ub-E7(49–57), pcDNA3-4Ub-E7(49–57), and pcDNA3-MITD-E7(49–57), respectively. The method for generating pcDNA3-CRT-E7(49–57) is mentioned previously. Antigen presentation of HPV16 E7 was evaluated by transfected DC2.4. DC2.4 were transfected with 1 ug of (1) pcDNA3 vector, (2) pcDNA3-1Ub-E7(49–57), (3) pcDNA3-4Ub-E7(49–57), (4) pcDNA3-E7(49–57), (5) pcDNA3-MITD-E7(49–57), or (6) pcDNA3-CRT-E7(49–57), and then co-cultured with HPV16 E7aa49-57 peptide-specific CD8⁺ T cell line. Following incubation, the E7-specific T cells were stained for surface CD8 and intracellular IFN-γ, then analyzed by flow cytometry. (**b**) Representative flow cytometry plot illustrating the amount of E7-specific CD8⁺ IFN-γ⁺ T cells (**c**) Bar graph summary of the flow cytometry results. Data are presented as mean ± SD of three biological replicates and analyzed using one-way ANOVA (**c**). ****p < .0001, ns: not significant.

**Fig. 6:**
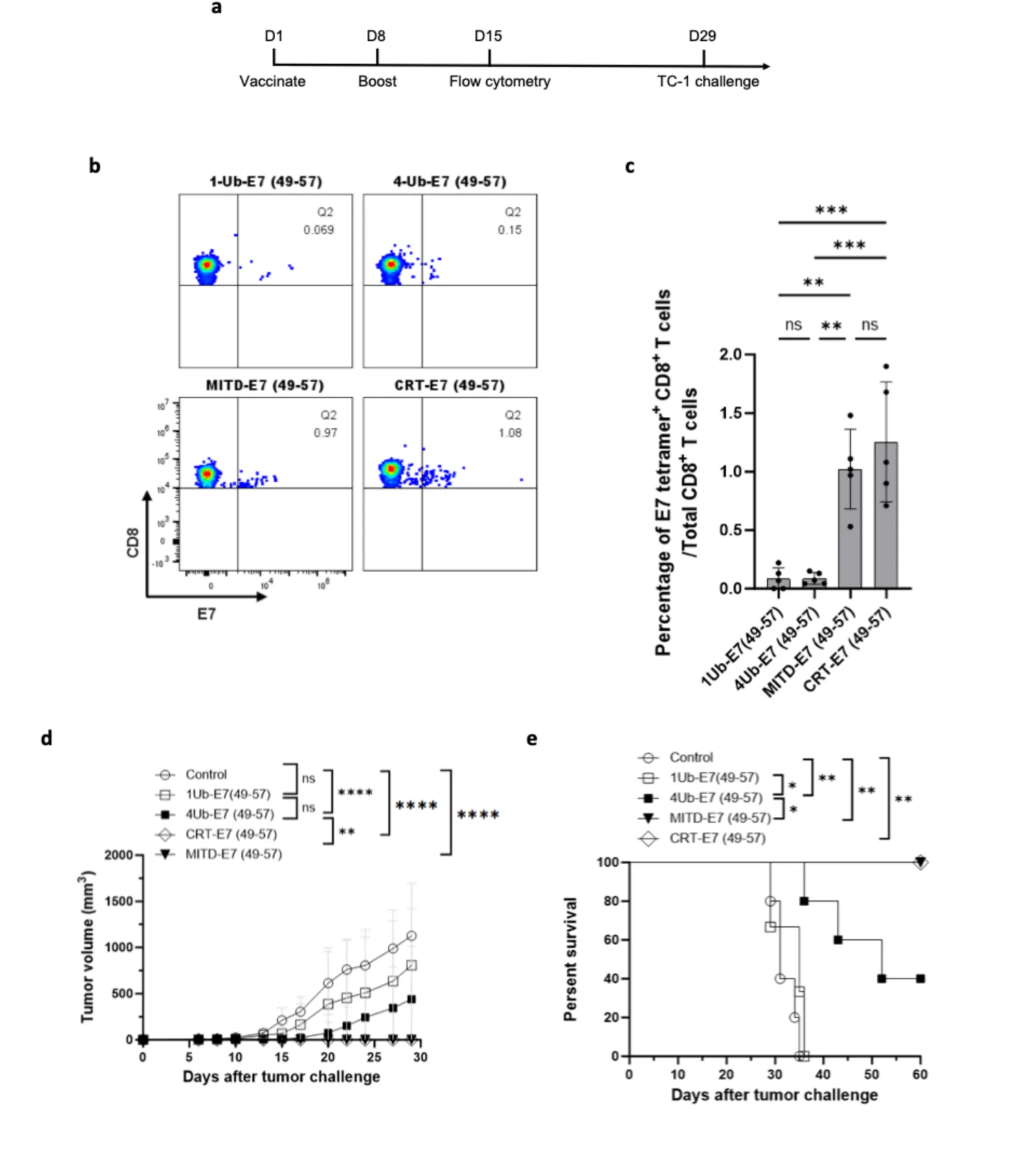
Comparison of in vivo protection efficacy of different potent constructs (**a**) Timeline of the experiment. Briefly, on day 1, mice (*n* = 5 per group) were immunized intramuscularly with (1) 10 µg of pcDNA3-CRT-E7(49–57), (2) 10 µg of pcDNA3-1Ub-E7(49–57), (3) 10 µg of pcDNA-4Ub-E7(49–57) or (4) 10 µg of pcDNA-MITD-E7(49–57) followed by electroporation. On day 8, the mice were boosted using the same regimen. On day 15, PBMCs were collected and analyzed via flow cytometry. On day 29, mice were subcutaneously challenged with 1 × 10^5^ TC-1 tumor cells. Tumor size and survival were subsequently monitored. (**b**) Representative flow cytometry plot showing the frequency of E7-specific CD8⁺ T cells within the CD8⁺ T cell population. (**c**) Bar graph summarizing the quantitative results of the flow cytometry analysis. (**d**) Line graph illustrating tumor volume progression in TC-1 tumor-challenged mice (*n* = 3 for 1-Ub). Two mice died early after tumor challenge before tumors became measurable. These deaths were unlikely to be related to tumor progression and were therefore excluded. (**e**) Kaplan-Meier analysis was used to assess the 60-day survival of mice bearing TC-1 tumors. Data are presented as mean ± SD and analyzed by repeated measures ANOVA (**c**) and Kaplan-Meier method with Log-rank (Mantel-Cox) test (**c**). *p < .05, **p < .01, ***p < .001, ****p < .0001, ns: not significant.

To compare the efficacy of generating E7-specific immune responses with other established DNA vaccine strategies that enhance antigen presentation, we first generated Ub-E7(49–57) (epitope-specific Ub-E7) and MITD-E7(49–57) (epitope-specific MITD-E7) constructs using the same fusion strategy as for generating CRT-E7(49–57) (Fig. 5a). To assess E7 epitope presentation, DC2.4 cells were transfected with either E7(49–57), CRT-E7(49–57), 1Ub-E7(49–57), 4Ub-E7(49–57), MITD-E7(49–57) or empty pcDNA3 vector, and then co-cultured with an E7-specific CD8^+^ T cell line. E7-specific cytotoxic T lymphocytes (CTL) activation was measured to reflect E7 presentation by staining for surface CD8 and intracellular IFNγ and analyzed by flow cytometry. As shown in Fig. 5b, c, DC2.4 transfected with CRT-E7(49–57) had significantly higher levels of E7 epitope presentation compared to other strategies. In contrast, DC2.4 transfected with E7(49–57) and MITD presented at lower levels, while those transfected with the empty pcDNA3 vector and Ub-E7(49–57) exhibited similarly lowest presentation compared to other groups, resembling the natural presentation efficiency of E7 minigene (Fig. 5b, c). This provided in vitro evidence that CRT-E7(49–57) enhanced E7 antigen presentation. To compare the in vivo protection efficacy of these constructs against TC-1 tumors, mice were vaccinated intramuscularly with either CRT-E7(49–57), 1Ub-E7(49–57), 4Ub-E7(49–57), or MITD-E7(49–57), followed by electroporation. Immunizations and tumor implantation were carried out following the schedule outlined in Fig. 6a. Seven days following the final vaccination, PBMCs were collected and analyzed via flow cytometry. Mice vaccinated with 1Ub-E7(49–57) and 4Ub-E7(49–57) induced very weak E7-specific CD8^+^ T cell responses with no significant difference between them, whereas those vaccinated with CRT-E7(49–57) and MITD-E7(49–57) had robust responses, with CRT-E7(49–57) showing a slightly higher average percentage (Fig. 6b, c). Consistent with the immune response data, mice receiving CRT-E7(49–57) or MITD-E7(49–57) remained tumor-free for one month and had longer survival compared to other groups. These results suggest that the CRT-E7(49–57) vaccine elicits stronger immune responses and provides protection comparable to MITD-fused vaccines and superior to Ub-fused vaccines.

### CRT-E7(49–57) exhibits enhanced therapeutic antitumor effects than Ub-E7(49–57) and MITD-E7(49–57)

**Fig. 7:**
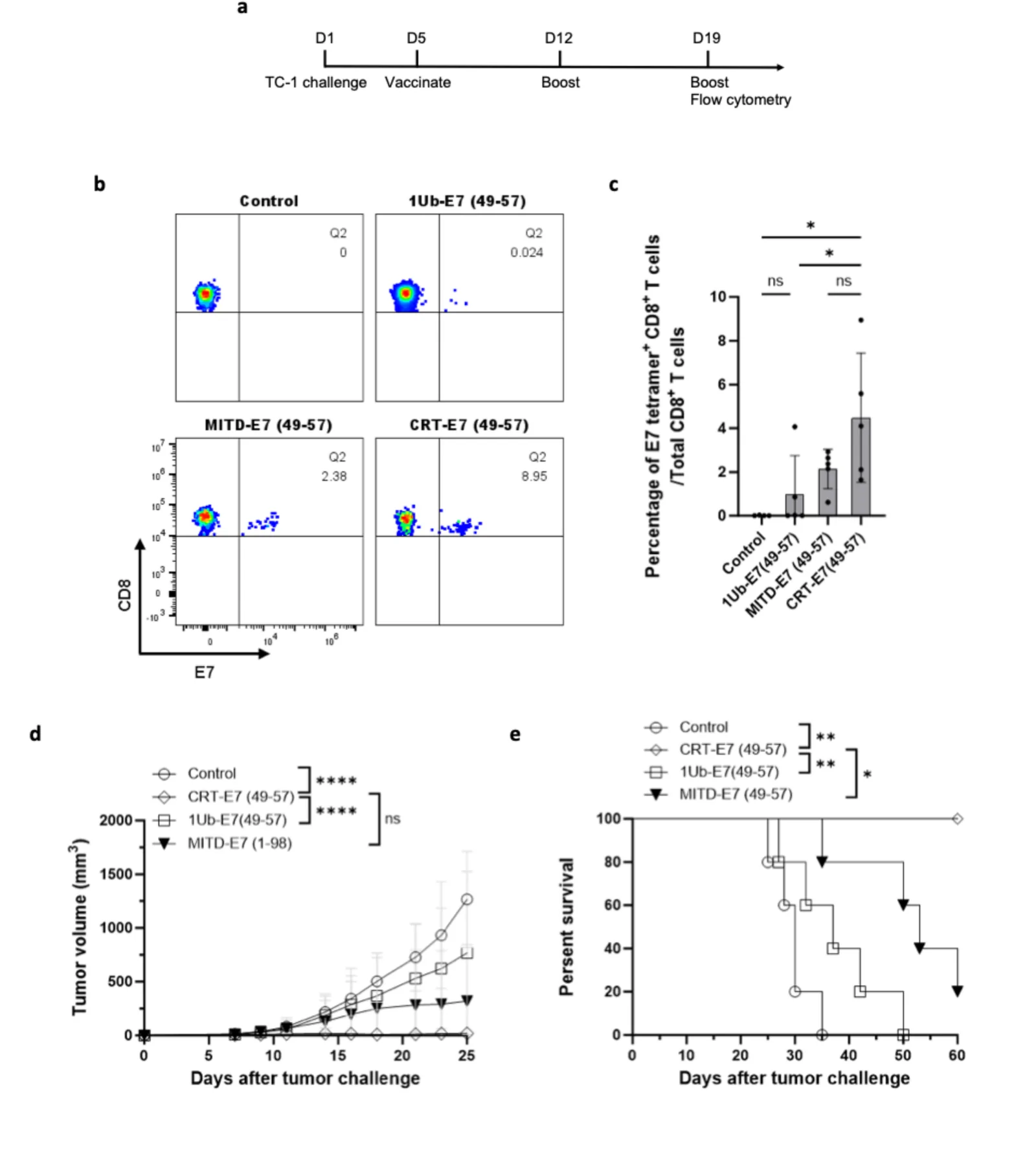
Comparison of in vivo treatment efficacy of different potent constructs. (**a**) Timeline of experiment. Briefly, on day 1, mice (*n* = 5 per group) were challenged subcutaneously with 1 × 10^5^ TC-1 tumor cells. On day 5, mice were immunized intramuscularly with (1) control, no treatment, (2) 10 µg of pcDNA3-1-Ub-E7(49–57), (3) 10 µg of pcDNA3-MITD-E7(49–57), or (4) 10 µg of pcDNA3-CRT-E7(49–57), followed by electroporation. On days 12 and 19, the mice received booster doses following the same regimen. On day 19, flow cytometry was performed on PBMCs to evaluate E7-specific T cell responses. (**b**) Representative flow cytometry plot showing the frequency of E7-specific CD8⁺ T cells within the CD8⁺ T cell population. (**c**) Bar graph summarizing the quantitative results of the flow cytometry analysis. (**d**) Line graph illustrating tumor volume progression in TC-1 tumor-challenged mice. (**e**) Kaplan-Meier analysis was used to assess the 60-day survival of mice bearing TC-1 tumors. Data are presented as mean ± SD and analyzed by repeated measures ANOVA (**c**) and Kaplan-Meier method with Log-rank (Mantel-Cox) test (**c**). *p < .05, **p < .01, ****p < .0001, ns: not significant.

We next evaluated the therapeutic potential of CRT-E7(49–57) in mice bearing TC-1 tumors, alongside Ub-E7(49–57) and MITD-E7(49–57) vaccines. Tumor implantation, immunizations and flow cytometry analysis were conducted according to the schedule shown in Fig. 7a. Flow cytometry analysis revealed that mice vaccinated with CRT-E7(49–57) and MITD-E7(49–57) exhibited robust E7-specific CD8⁺ T cell responses, whereas Ub-E7(49–57) and the control group showed minimal responses (Fig. 7b, c). Consistent with the immune response data and our expectation, mice receiving CRT-E7(49–57) remained tumor-free for 25 days and exhibiting 100% survival for over 60 days (Fig. 7d, e). In contrast, MITD-E7(49–57) demonstrated reduced therapeutic efficacy in terms of tumor progression and survival, while 1Ub-E7(49–57) showed the least efficacy among the tested groups (Fig. 7e). These findings demonstrate that the CRT-E7(49–57) vaccine is the most effective among the tested strategies in the in vivo therapeutic model, highlighting its promising therapeutic potential.

## Discussion

In this study, we demonstrated that the CRT-fused HPV-16 E7 minigene vaccine CRT-E7(49–57) elicited robust E7-specific CD8⁺ T cell responses. We focused on CD8⁺ T cell response because we previously demonstrated that the antitumor activity of CRT-linked DNA vaccines in the TC-1 tumor model is independent of CD4⁺ T cells and NK cells^19^. Notably, the responses were comparable to those induced by the CRT-E7(1–98). In both prophylactic and therapeutic models, mice immunized with the CRT-E7(49–57) vaccine developed significantly reduced tumor burden following tumor challenge compared with control groups and survived for up to two months. Furthermore, after tumor rechallenge, vaccinated mice maintained high levels of E7-specific CD8⁺ T cells and exhibited markedly improved survival, suggesting the induction of sustained immune memory in vivo. The enhancement of E7-specific CD8⁺ T cell responses might be due to the ER-chaperone effect of CRT, which facilitated antigen processing, loading, presentation, and ultimately the generation of antigen-specific CD8⁺ T precursors.

To further assess the efficacy CRT-E7(49–57), we compared its performance with two established approaches that also enhance antigen presentation but via distinct mechanisms: Ub-E7(49–57) and MITD-E7(49–57). Ub linkage enhances antigen presentation by accelerating intracellular proteasome-mediated antigen degradation, whereas MITD linkage enhances antigen presentation by mimicking the natural trafficking of MHC molecules. The results showed that CRT fusion induced a significantly stronger E7-specific CD8⁺ T cell response than Ub-linked vaccination, with no significant difference compared to the MITD-E7(49–57) vaccine. Consistently, the CRT-E7(49–57) group demonstrated comparable tumor suppression and improved survival in comparison to the group treated with the MITD-E7(49–57), supporting its potential as a promising DNA vaccine platform for enhancing immune responses and improving cancer treatment.

Several studies have reported that the Ub-fusion strategy does not consistently enhance antigen-specific CD8⁺ T cell responses, which might explain why Ub-E7 construct was ineffective in our case^40,43,44^. The effectiveness of Ub-fusion heavily depends on the intrinsic properties of the antigen^52–54^. Certain proteins, such as lymphocytic choriomeningitis virus nucleoprotein, respond well to Ub-fusion^43^, whereas others such as influenza nucleoprotein do not^55^. This indicates that fusing Ub to an antigen is not universally beneficial for antigen processing. Moreover, in the absence of in-depth conformational analyses, it remains uncertain whether the expressed Ub-E7 retains its native conformation. In the case that Ub-fusion results in a non-native or misfolded structure, the antigen may not be efficiently processed for MHC I presentation. Another possible explanation is that although Ub-fusion accelerates antigen degradation, this does not necessarily enhance antigen presentation^56,57^. This is supported by Fig. 5c, only a slight increase in E7 presentation was observed for the Ub-E7(49–57) construct in the in vitro assay. Studies showed that antigen fused with MITD along with a signal peptide leads to enhanced MHC I epitope presentation in human and murine dendritic cells (DCs)^38^. In DNA vaccine models, immunogenicity largely depends on cross-presentation pathways to initiate effective immune responses^58,59^. Mechanistically, newly synthesized polypeptides are translocated into the ER, where they undergo processing and maturation. MITD sequences facilitate the targeting of these processed peptides to specific intracellular compartments, including the ER, Golgi apparatus, and endosomes, resulting in efficient presentation of cytotoxic T cell epitope^60^. The enhanced immunogenicity observed with MITD-E7 is therefore likely due to MITD-mediated intracellular trafficking in both immature and mature DCs, which improves antigen processing and augments cross-presentation efficiency^61^.

In our study, the precise mechanism by which CRT enhances E7-specific CD8⁺ T cell responses remains to be fully elucidated. One plausible explanation is that CRT directs antigen into the ER lumen, facilitating more efficient MHC class I peptide loading. As an ER-resident chaperone, CRT may also stabilize peptide-loading complexes, promoting optimal peptide-MHCI assembly. Additionally, surface-exposed CRT on apoptotic cells can act as an “eat-me” signal, enhancing dendritic cell phagocytosis and cross-presentation, thereby boosting cellular immunity^1,62^. Another potential mechanism involves ER-associated degradation (ERAD), whereby misfolded or improperly assembled proteins are recognized by ER chaperones, unfolded, and retrotranslocated to the cytosol for proteasomal degradation. CRT participates in the early recognition and unfolding steps of ERAD^63^, and elevated CRT levels may increase the pool of peptides available for MHCI presentation. While the exact reason for the improved survival observed with CRT-E7(49–57) compared to MITD-E7(49–57) remains unclear, these findings collectively suggest that CRT fusion enhances overall MHCI antigen presentation. Further studies examining proteasome dependence, CRT expression level, ER trafficking dynamics, and stabilization of the PLC are necessary to delineate the underlying mechanisms.

DNA vaccines face several challenges in clinical translation. In addition to concerns about relatively low immunogenicity, they are subject to stringent regulatory guidelines due to the theoretical risk of genomic integration, which complicates their approval for human use^64^. Nevertheless, DNA vaccines represent a promising strategy for cancer immunotherapy with advantages such as easy production and storage, low cost, high stability, capacity to accommodate multiple sequences, and the ability to stimulate both cellular and humoral immunity. These features make them particularly valuable for developing poly-epitope vaccines that could elicit durable T cell responses against tumor-specific neoantigens. The increasing precision of tumor sequencing and bioinformatics has further advanced this field by enabling the rapid identification of patient-specific neoantigens, thus enhancing the potential of DNA vaccines to deliver personalized immunotherapy across diverse cancer types^65^. However, generating sufficient immune responses often requires additional strategies to enhance immunogenicity (e.g. adjuvants). One solution has been the incorporation of fusion partners such as Ub, lysosomal signals, or CRT to facilitate antigen presentation^15^. Among these, CRT fusion has demonstrated to be exceptionally effective, as our previous studies revealed that coupling CRT to DNA-based vaccines significantly improves therapeutic outcomes across diverse antigen combinations^19,66,67^. Although theoretical concerns remain regarding genomic insertion, clinical trials have shown such events to be rare, reflecting safety of DNA-based vaccination^65,68^. Notably, RNA vaccines also hold strong promise in terms of safety and efficacy, as mRNA-based strategies have already shown encouraging results in targeting various diseases^69^. Accordingly, there is considerable motivation to expand the CRT platform to RNA vaccines for cancer treatment.

Immunotherapy plays a crucial role in the ongoing battle against cancer. However, tumors can develop resistance, allowing them to evade immune surveillance and withstand therapeutic interventions^70,71^. While the CRT fusion technique generates a promising immune response against neoantigens, combining it with other immunotherapy techniques may be necessary to overcome resistance. Studies suggest that incorporating multiple strategies can augment anti-tumor efficacy^72,73^, including chemotherapy, radiotherapy, and immune checkpoint therapy. Further research is needed to explore effective combination therapy involving CRT.

In conclusion, our results demonstrate that linking CRT to HPV-16 E7 minigene can produce profound antitumor efficacy in TC-1 murine tumor models by boosting E7-specific CD8⁺ T cell responses. Importantly, it achieves efficacy comparable to full-length E7 while outperforming conventional approaches. This fusion strategy could potentially be adapted for RNA vaccines, combined with other immunotherapeutic approaches, and applied to cancer systems beyond TC-1.

## Supplementary Information

Below is the link to the electronic supplementary material.

Supplementary Material 1

Supplementary Material 2

## Data Availability

All data relevant to the study are included in the article or uploaded as Additional information. Data and materials are available on reasonable request.

## Abbreviations

CRT: Calreticulin
CTL: Cytotoxic T Lymphocytes
ER: Endoplasmic Reticulum
MHCI: Major Histocompatibility Complex Class I
MITD: MHCI Trafficking Domain
PLC: Peptide Loading Complex
TAA: Tumor Associated Antigen
TSA: Tumor Specific Antigen
Ub: Ubiquitin

## References

[1] Raghavan, M., Wijeyesakere, S. J., Peters, L. R. & Del Cid, N. Calreticulin in the immune system: ins and outs. *Trends Immunol.* **34** (1), 13–21 (2013). 10.1016/j.it.2012.08.002 PMC4117402 22959412

[2] Nash, P. D., Opas, M. & Michalak, M. Calreticulin: not just another calcium-binding protein. *Mol. Cell. Biochem.* **135** (1), 71–78 (1994). 10.1007/BF00925962 7816058

[3] Fucikova, J., Spisek, R., Kroemer, G. & Galluzzi, L. Calreticulin and cancer. *Cell. Res.* **31** (1), 5–16 (2021). 10.1038/s41422-020-0383-9 PMC7853084 32733014

[4] Blum, J. S., Wearsch, P. A. & Cresswell, P. Pathways of antigen processing. *Annu. Rev. Immunol.* **31**, 443–473 (2013). 10.1146/annurev-immunol-032712-095910 PMC4026165 23298205

[5] Domnick, A. et al. Molecular basis of MHC I quality control in the peptide loading complex. *Nat. Commun.* **13** (1), 4701 (2022). 10.1038/s41467-022-32384-z PMC9365787 35948544

[6] Spee, P. & Neefjes, J. TAP-translocated peptides specifically bind proteins in the endoplasmic reticulum, including gp96, protein disulfide isomerase and calreticulin. *Eur. J. Immunol.* **27** (9), 2441–2449 (1997 Sept). 10.1002/eji.1830270944 9341791

[7] Gao, B. et al. Assembly and Antigen-Presenting Function of MHC Class I Molecules in Cells Lacking the ER Chaperone Calreticulin. *Immunity* **16** (1), 99–109 (2002). 10.1016/s1074-7613(01)00260-6 11825569

[8] Zaidi, N., Jaffee, E. M. & Yarchoan, M. Recent advances in therapeutic cancer vaccines. *Nat. Rev. Cancer*. **25** (7), 517–533 (2025 July). 10.1038/s41568-025-00820-z 40379970

[9] Hu, Z., Ott, P. A. & Wu, C. J. Towards personalized, tumour-specific, therapeutic vaccines for cancer. *Nat. Rev. Immunol.* **18** (3), 168–182 (2018). 10.1038/nri.2017.131 PMC6508552 29226910

[10] Katsikis, P. D., Ishii, K. J. & Schliehe, C. Challenges in developing personalized neoantigen cancer vaccines. *Nat. Rev. Immunol.* **24** (3), 213–227 (2024). 10.1038/s41577-023-00937-y PMC12001822 37783860

[11] Blass, E. & Ott, P. A. Advances in the development of personalized neoantigen-based therapeutic cancer vaccines. *Nat. Rev. Clin. Oncol.* **18** (4), 215–229 (2021). 10.1038/s41571-020-00460-2 PMC7816749 33473220

[12] Lang, F., Schrörs, B., Löwer, M., Türeci, Ö. & Sahin, U. Identification of neoantigens for individualized therapeutic cancer vaccines. *Nat. Rev. Drug Discov*. **21** (4), 261–282 (2022). 10.1038/s41573-021-00387-y PMC7612664 35105974

[13] Lu, B. et al. The next-generation DNA vaccine platforms and delivery systems: advances, challenges and prospects. *Front. Immunol.* **15**, 1332939 (2024). 10.3389/fimmu.2024.1332939 PMC10867258 38361919

[14] Neeli, P., Chai, D., Roy, D., Prajapati, S. & Bonam, S. R. DNA Vaccines in the Post-mRNA Era: Engineering, Applications, and Emerging Innovations. *Int. J. Mol. Sci. 2025 Sept* **7**;26(17):8716 . 10.3390/ijms26178716 PMC12429263 40943636

[15] Liao, H. C. & Liu, S. J. Advances in nucleic acid-based cancer vaccines. *J. Biomed. Sci.* **32** (1), 10 (2025). 10.1186/s12929-024-01102-w PMC11748563 39833784

[16] Aggarwal, C. et al. Safety and Efficacy of MEDI0457 plus Durvalumab in Patients with Human Papillomavirus-Associated Recurrent/Metastatic Head and Neck Squamous Cell Carcinoma. *Clin. Cancer Res. Off J. Am. Assoc. Cancer Res.* **29** (3), 560–570 (2023). 10.1158/1078-0432.CCR-22-1987 PMC9890138 36455147

[17] Trimble, C. L. et al. Safety, efficacy, and immunogenicity of VGX-3100, a therapeutic synthetic DNA vaccine targeting human papillomavirus 16 and 18 E6 and E7 proteins for cervical intraepithelial neoplasia 2/3: a randomised, double-blind, placebo-controlled phase 2b trial. *Lancet Lond. Engl.* **386** (10008), 2078–2088 (2015). 10.1016/S0140-6736(15)00239-1 PMC4888059 26386540

[18] Youn, J. W. et al. Pembrolizumab plus GX-188E therapeutic DNA vaccine in patients with HPV-16-positive or HPV-18-positive advanced cervical cancer: interim results of a single-arm, phase 2 trial. *Lancet Oncol.* **21** (12), 1653–1660 (2020). 10.1016/S1470-2045(20)30486-1 33271094

[19] Cheng, W. F. et al. Tumor-specific immunity and antiangiogenesis generated by a DNA vaccine encoding calreticulin linked to a tumor antigen. *J. Clin. Invest.* **108** (5), 669–678 (2001 Sept). 10.1172/JCI12346 PMC209378 11544272

[20] Vigneron, N. & Van den Eynde, B. J. Proteasome subtypes and regulators in the processing of antigenic peptides presented by class I molecules of the major histocompatibility complex. *Biomolecules* **4** (4), 994–1025 (2014). 10.3390/biom4040994 PMC4279167 25412285

[21] Li, L. et al. Engineering superior DNA vaccines: MHC class I single chain trimers bypass antigen processing and enhance the immune response to low affinity antigens. *Vaccine* **28** (8), 1911–1918 (2010). 10.1016/j.vaccine.2009.10.096 PMC2830906 20188246

[22] Aurisicchio, L. et al. A novel minigene scaffold for therapeutic cancer vaccines. *Oncoimmunology* **3** (1), e27529 (2014). 10.4161/onci.27529 PMC4002591 24790791

[23] Li, J., Ju, Y., Jiang, M., Li, S. & Yang, X. Y. Epitope-Based Vaccines: The Next Generation of Promising Vaccines Against Bacterial Infection. *Vaccines* **13** (3), 248 (2025). 10.3390/vaccines13030248 PMC11946261 40266107

[24] Li, L. et al. Optimized polyepitope neoantigen DNA vaccines elicit neoantigen-specific immune responses in preclinical models and in clinical translation. *Genome Med.* **13** (1), 56 (2021). 10.1186/s13073-021-00872-4 PMC8059244 33879241

[25] Guo, N. et al. Immunoinformatics Design and In Vivo Immunogenicity Evaluation of a Conserved CTL Multi-Epitope Vaccine Targeting HPV16 E5, E6, and E7 Proteins. *Vaccines* **12** (4), 392 (2024). 10.3390/vaccines12040392 PMC11053576 38675774

[26] Peng, K., Zhao, X., Fu, Y. X. & Liang, Y. Eliciting antitumor immunity via therapeutic cancer vaccines. *Cell. Mol. Immunol. 2025 July* **9**;22(8):840–868 . 10.1038/s41423-025-01316-4 PMC12311208 40629076

[27] Kenter, G. G. et al. Vaccination against HPV-16 oncoproteins for vulvar intraepithelial neoplasia. *N Engl. J. Med.* **361** (19), 1838–1847 (2009). 10.1056/NEJMoa0810097 19890126

[28] Rosalia, R. A. et al. Dendritic cells process synthetic long peptides better than whole protein, improving antigen presentation and T-cell activation. *Eur. J. Immunol.* **43** (10), 2554–2565 (2013). 10.1002/eji.201343324 23836147

[29] Massarelli, E. et al. Combining Immune Checkpoint Blockade and Tumor-Specific Vaccine for Patients With Incurable Human Papillomavirus 16-Related Cancer: A Phase 2 Clinical Trial. *JAMA Oncol.* **5** (1), 67–73 (2019). 10.1001/jamaoncol.2018.4051 PMC6439768 30267032

[30] Ramos da Silva, J. et al. Single immunizations of self-amplifying or non-replicating mRNA-LNP vaccines control HPV-associated tumors in mice. *Sci. Transl Med.* **15** (686), eabn3464 (2023). 10.1126/scitranslmed.abn3464 36867683

[31] Kanuma, T. et al. CD63-Mediated Antigen Delivery into Extracellular Vesicles via DNA Vaccination Results in Robust CD8 + T Cell Responses. *J. Immunol. Baltim. Md.* **2017** (12), 4707–4715 (1950). 10.4049/jimmunol.1600731 28507029

[32] El Safadi, D. et al. Exosome-Mediated Antigen Delivery: Unveiling Novel Strategies in Viral Infection Control and Vaccine Design. *Vaccines* **12** (3), 280 (2024). 10.3390/vaccines12030280 PMC10974137 38543914

[33] He, J. et al. Tuning the fluidity and protein corona of ultrasound-responsive liposomal nanovaccines to program T cell immunity in mice. *Nat. Commun. 2024 Sept* **16**;15(1):8121 . 10.1038/s41467-024-52104-z PMC11405680 39284814

[34] Lin, C. T. et al. Enhancement of DNA vaccine potency through linkage of antigen gene to ER chaperone molecules, ER-60, tapasin, and calnexin. *J. Biomed. Sci.* **12** (2), 279–287 (2005). 10.1007/s11373-005-0334-y 15918000

[35] Tominaga, S. et al. Induced pluripotent stem cell-derived dendritic cell vaccine therapy genetically modified on the ubiquitin-proteasome system. *Gene Ther.* **30** (7–8), 552–559 (2023). 10.1038/s41434-023-00388-z 36959396

[36] Huebener, N. et al. Vaccination with minigenes encoding for novel self antigens are effective in DNA-vaccination against neuroblastoma. *Cancer Lett.* **197** (1-2), 211–217 (2003). July 18. 10.1016/s0304-3835(03)00102-2 12880984

[37] Luozhong, S. et al. Poly(carboxybetaine) lipids enhance mRNA therapeutics efficacy and reduce their immunogenicity. Nat Mater [Internet]. May 29 [cited 2025 Oct 1]; (2025). Available from: https://www.nature.com/articles/s41563-025-02240-8 10.1038/s41563-025-02240-8 PMC12354250 40442447

[38] Kreiter, S. et al. Increased antigen presentation efficiency by coupling antigens to MHC class I trafficking signals. *J. Immunol. Baltim. Md. 1950*. **180** (1), 309–318 (2008). 10.4049/jimmunol.180.1.309 18097032

[39] Sahin, U. et al. Personalized RNA mutanome vaccines mobilize poly-specific therapeutic immunity against cancer. *Nat. 2017 July* **13**;547(7662):222–226 . 10.1038/nature23003 28678784

[40] Husnjak, K. & Dikic, I. Ubiquitin-binding proteins: decoders of ubiquitin-mediated cellular functions. *Annu. Rev. Biochem.* **81**, 291–322 (2012). 10.1146/annurev-biochem-051810-094654 22482907

[41] Leone, P. et al. MHC class I antigen processing and presenting machinery: organization, function, and defects in tumor cells. *J. Natl. Cancer Inst.* **105** (16), 1172–1187 (2013). 10.1093/jnci/djt184 23852952

[42] Andersson, H. A. & Barry, M. A. Maximizing antigen targeting to the proteasome for gene-based vaccines. *Mol. Ther. J. Am. Soc. Gene Ther.* **10** (3), 432–446 (2004 Sept). 10.1016/j.ymthe.2004.05.035 15336644

[43] Rodriguez, F., Zhang, J. & Whitton, J. L. DNA immunization: ubiquitination of a viral protein enhances cytotoxic T-lymphocyte induction and antiviral protection but abrogates antibody induction. *J. Virol.* **71** (11), 8497–8503 (1997). 10.1128/jvi.71.11.8497-8503.1997 PMC192313 9343207

[44] Vidalin, O., Tanaka, E., Spengler, U., Trépo, C. & Inchauspé, G. Targeting of hepatitis C virus core protein for MHC I or MHC II presentation does not enhance induction of immune responses to DNA vaccination. *DNA Cell. Biol.* **18** (8), 611–621 (1999). 10.1089/104454999315024 10463057

[45] Kranz, L. M. et al. Systemic RNA delivery to dendritic cells exploits antiviral defence for cancer immunotherapy. *Nat. 2016 June* **16**;534(7607):396–401 . 10.1038/nature18300 27281205

[46] Yang, A. et al. Enhancing antitumor immunogenicity of HPV16-E7 DNA vaccine by fusing DNA encoding E7-antigenic peptide to DNA encoding capsid protein L1 of Bovine papillomavirus. *Cell. Biosci.* **7**, 46 (2017). 10.1186/s13578-017-0171-5 PMC5569540 28852471

[47] Dantuma, N. P., Lindsten, K., Glas, R., Jellne, M. & Masucci, M. G. Short-lived green fluorescent proteins for quantifying ubiquitin/proteasome-dependent proteolysis in living cells. *Nat. Biotechnol.* **18** (5), 538–543 (2000). 10.1038/75406 10802622

[48] Lin, K. Y. et al. Treatment of established tumors with a novel vaccine that enhances major histocompatibility class II presentation of tumor antigen. *Cancer Res.* **56** (1), 21–26 (1996). 8548765

[49] Shen, Z., Reznikoff, G., Dranoff, G. & Rock, K. L. Cloned dendritic cells can present exogenous antigens on both MHC class I and class II molecules. J Immunol Baltim Md. 1997;158(6):2723–30. (1950). 9058806

[50] Wang, T. L. et al. Intramuscular administration of E7-transfected dendritic cells generates the most potent E7-specific anti-tumor immunity. *Gene Ther.* **7** (9), 726–733 (2000). 10.1038/sj.gt.3301160 10822298

[51] Peng, S. et al. Identification of the murine H-2D(b) and human HLA-A*0201 MHC class I-restricted HPV6 E7-specific cytotoxic T lymphocyte epitopes. *Cancer Immunol. Immunother CII*. **65** (3), 261–271 (2016). 10.1007/s00262-016-1793-x PMC5690558 26759151

[52] Michalek, M. T., Grant, E. P., Gramm, C., Goldberg, A. L. & Rock, K. L. A role for the ubiquitin-dependent proteolytic pathway in MHC class l-restricted antigen presentation. *Nat. 1993 June* **10**;363(6429):552–554 . 10.1038/363552a0 8389422

[53] Leachman, S. A. et al. Ubiquitin-Fused and/or Multiple Early Genes from Cottontail Rabbit Papillomavirus as DNA Vaccines. *J. Virol.* **76** (15), 7616–7624 (2002). 10.1128/JVI.76.15.7616-7624.2002 PMC136350 12097575

[54] Tobery, T. W. & Siliciano, R. F. Targeting of HIV-1 Antigens for Rapid Intracellular Degradation Enhances Cytotoxic T Lymphocyte (CTL) Recognition and the Induction of De Novo CTL Responses In Vivo After Immunization. *J. Exp. Med.* **185** (5), 909–920 (1997). 10.1084/jem.185.5.909 PMC2196169 9120397

[55] Fu, T. Induction of MHC class I-restricted CTL response by DNA immunization with ubiquitin-influenza virus nucleoprotein fusion antigens. *Vaccine* **16** (18), 1711–1717 (1998). 10.1016/s0264-410x(98)00134-0 9778746

[56] Huang, L., Marvin, J. M., Tatsis, N. & Eisenlohr, L. C. Cutting Edge: Selective role of ubiquitin in MHC class I antigen presentation. *J. Immunol. Baltim. Md. 1950*. **186** (4), 1904–1908 (2011). 10.4049/jimmunol.1003411 PMC3650468 21239720

[57] Goth, S., Nguyen, V. & Shastri, N. Generation of naturally processed peptide/MHC class I complexes is independent of the stability of endogenously synthesized precursors. J Immunol Baltim Md. 1996 Sept 1;157(5):1894–904. (1950). 8757307

[58] Bolhassani, A. & Yazdi, S. R. DNA immunization as an efficient strategy for vaccination. *Avicenna J. Med. Biotechnol.* **1** (2), 71–88 (2009 July). PMC3558129 23407787

[59] Shirota, H., Petrenko, L., Hong, C. & Klinman, D. M. Potential of transfected muscle cells to contribute to DNA vaccine immunogenicity. J Immunol Baltim Md. 2007 July 1;179(1):329–36. (1950). 10.4049/jimmunol.179.1.329 17579053

[60] Zhang, Y. et al. MHCI trafficking signal-based mRNA vaccines strengthening immune protection against RNA viruses. *Bioeng. Transla Med.* **10**, e10709 (2025). 10.1002/btm2.10709 PMC11711215 39801759

[61] Cao, S. et al. A DNA Vaccine Incorporating the MHC Class I Trafficking Domain and PADRE Epitope Enhances Antitumor Immunity in a Murine Pancreatic Cancer Model. *IJMS* **27**, 2039 (2026). 10.3390/ijms27042039 PMC12940583 41752180

[62] Ye, X., Li, W., Huang, J., Zhang, L. & Zhang, Y. Cytotoxic T Cell Responses Induced by CS1/CRT Fusion DNA Vaccine in a Human Plasmacytoma Model. *Front. Oncol.* **10**, 587237 (2020). 10.3389/fonc.2020.587237 PMC7714938 33330069

[63] Williams, D. B. Beyond lectins: the calnexin/calreticulin chaperone system of the endoplasmic reticulum. *J. Cell Sci.* **119**, 615–623 (2006). 10.1242/jcs.02856 16467570

[64] Zhou, Z. P. et al. DNA-Based Vaccines: Advances, Applications, and Future Prospects. Genes & Diseases. ;102025. (2026). 10.1016/j.gendis.2025.102025 PMC13375960 42491006

[65] Konopka, E. N., Edgerton, A. O. & Kutzler, M. A. Nucleic acid vaccines: innovations, efficacy, and applications in at-risk populations. *Front. Immunol.* **16**, 1584876 (2025). 10.3389/fimmu.2025.1584876 PMC12116436 40438110

[66] Park, Y. S., Lee, J. H., Hung, C. F., Wu, T. C. & Kim, T. W. Enhancement of antibody responses to Bacillus anthracis protective antigen domain IV by use of calreticulin as a chimeric molecular adjuvant. *Infect. Immun.* **76** (5), 1952–1959 (2008). 10.1128/IAI.01722-07 PMC2346671 18285494

[67] Kim, T. W. et al. Generation and characterization of DNA vaccines targeting the nucleocapsid protein of severe acute respiratory syndrome coronavirus. *J. Virol.* **78** (9), 4638–4645 (2004). 10.1128/JVI.78.9.4638-4645.2004 PMC387705 15078946

[68] Disis, M. L. N. et al. Safety and Outcomes of a Plasmid DNA Vaccine Encoding the ERBB2 Intracellular Domain in Patients With Advanced-Stage ERBB2-Positive Breast Cancer: A Phase 1 Nonrandomized Clinical Trial. *JAMA Oncol.* **9** (1), 71–78 (2023). 10.1001/jamaoncol.2022.5143 PMC9634596 36326756

[69] Rosa, S. S., Prazeres, D. M. F., Azevedo, A. M. & Marques, M. P. C. mRNA vaccines manufacturing: Challenges and bottlenecks. *Vaccine* **39** (16), 2190–2200 (2021). 10.1016/j.vaccine.2021.03.038 PMC7987532 33771389

[70] Bell, H. N. & Zou, W. Beyond the Barrier: Unraveling the Mechanisms of Immunotherapy Resistance. *Annu. Rev. Immunol.* **42** (1), 521–550 (2024 June). 10.1146/annurev-immunol-101819-024752 PMC11213679 38382538

[71] Kundu, M. et al. Modulation of the tumor microenvironment and mechanism of immunotherapy-based drug resistance in breast cancer. *Mol. Cancer*. **23** (1), 92 (2024). 10.1186/s12943-024-01990-4 PMC11075356 38715072

[72] Heinhuis, K. M. et al. Enhancing antitumor response by combining immune checkpoint inhibitors with chemotherapy in solid tumors. *Ann. Oncol. Off J. Eur. Soc. Med. Oncol.* **30** (2), 219–235 (2019). 10.1093/annonc/mdy551 30608567

[73] Meric-Bernstam, F., Larkin, J., Tabernero, J. & Bonini, C. Enhancing anti-tumour efficacy with immunotherapy combinations. *Lancet Lond. Engl.* **397** (10278), 1010–1022 (2021). 10.1016/S0140-6736(20)32598-8 33285141

