# Supplementary material for: A calreticulin-linked HPV-16 E7 minigene DNA vaccine elicits strong E7-specific CD8+ T-cell immunity and durable antitumor effects in a preclinical model
Source: Sci Rep. 2026 Jul 3;16:30645. doi: 10.1038/s41598-026-58415-z (PMC13630547; doi:10.1038/s41598-026-58415-z)
Supplement: Supplementary file 1 [file 41598_2026_58415_MOESM1_ESM.docx]

**Flow Cytometry Analysis and Gating Strategy for E7-Specific CD8+ T Cells**

To accurately quantify antigen-specific T cell responses, PBMCs were stained and analyzed using a rigorous sequential gating strategy to eliminate artifacts and non-specific signals. The detailed protocol is as follows:

**(1) Singlet Gating (SSC-A vs. SSC-Width):** To prevent false-positive signals from clumped cells, doublets were initially excluded. Single cells (Singlets) were stringently gated on an SSC-A (X-axis) versus SSC-Width (Y-axis) plot by delineating the primary linear population.

**(2) Lymphocyte Gating (FSC-A vs. SSC-A):** Following singlet selection, the target lymphocyte population was identified based on physical morphological properties. A morphological gate was applied using FSC-A (relative cell size) and SSC-A (internal granularity) to filter out smaller cell debris and larger, granular leukocyte subsets such as monocytes and granulocytes.

**(3) Live/Dead Gating (live_dead BV510-A vs. SSC-A):** Since dead cells exhibit non-specific antibody binding that increases background noise, live cells were selected. Dead cells were excluded by gating the negative population on the FL7-A :: live_dead BV510-A channel, with the threshold set strictly below the 10^4^ fluorescence intensity mark.

**(4) Effector Cell Identification (CD8 APC-A750-A vs. SSC-A):** Within the live lymphocyte gate, cytotoxic T cells were identified. The CD8+ T cell population was precisely gated by defining the positive population with a fluorescence intensity threshold of >10^4^ on the FL5-A :: CD8 APC-A750-A channel.

**(5) Antigen-Specific T Cell Quantification (E7 PE-A vs. CD8 APC-A750-A):** Finally, to evaluate the specific immune response, the E7-specific cells were quantified within the parental CD8+ T cell population. On a biaxial plot, quadrant gates were established with thresholds set at 10^4^ for both FL10-A :: E7 PE-A (X-axis) and FL5-A :: CD8 APC-A750-A (Y-axis). The E7 tetramer-positive cells were identified in quadrant 2 (Q2, PE >10^4 and APC-A750 >10^4).
